# Right heart failure due to compression of right ventricular outflow tract by post-operative aortic pseudoaneurysm

**DOI:** 10.1093/ehjcr/ytae024

**Published:** 2024-01-25

**Authors:** Shun Ijuin, Yushi Yamashita, Tamahiro Kinjo

**Affiliations:** Department of Cardiovascular Medicine, National Hospital Organization Kagoshima Medical Center: Kokuritsu Byoin Kiko Kagoshima Iryo Center, 8-1, Shiroyama-cho, Kagoshima-shi, Kagoshima 892-0853, Japan; Department of Cardiovascular Surgery, National Hospital Organization Kagoshima Medical Center: Kokuritsu Byoin Kiko Kagoshima Iryo Center, 8-1, Shiroyama-cho, Kagoshima-shi, Kagoshima 892-0853, Japan; Department of Cardiovascular Surgery, National Hospital Organization Kagoshima Medical Center: Kokuritsu Byoin Kiko Kagoshima Iryo Center, 8-1, Shiroyama-cho, Kagoshima-shi, Kagoshima 892-0853, Japan

A 78-year-old man presented with effort dyspnoea and oedema. He had undergone a replacement of ascending aorta for acute aortic dissection 3 years earlier, and no problems have been detected in post-operative computed tomography (CT). Echocardiogram showed a compressed right ventricle from extra-cardiac structures, resulting in right ventricular outflow tract (RVOT) stenosis and subsequent pulmonary hypertension (*Panel A*; Supplementary data online, *Video S1*). Computed tomography revealed a huge aortic pseudoaneurysm originating from peripheral anastomosis of the artificial aortic graft, which compressed the right ventricle as well as the superior vena cava, right atrium, and pulmonary artery (*Panels B* and *C*; Supplementary data online, *Video S2*). Additional reconstructed 3D-rendered CT clearly demonstrated that the RVOT was pinched and constricted by the aortic pseudoaneurysm (*Panels D* and *E*; Supplementary data online, *Video S3*). He was diagnosed with right heart failure due to compression around the RVOT by post-operative aortic pseudoaneurysm. He underwent a redo with a total arch replacement with an open stent graft to release the compression. During surgery, we found bleeding at the anastomosis between the graft and the aorta. We considered that the aortic tissue was fragile and the aortic wall would be dissected one after another by suturing haemostasis, so we added Bentall surgery. Post-operative computed tomography revealed the expanded RVOT (*Panel F*; Supplementary data online, *Video S4*) resulting in a haemodynamic normalization. He was discharged home 77 days after surgery. This is the first case of an aortic pseudoaneurysm that compressed the RVOT and led to a right heart failure.

**Figure:**
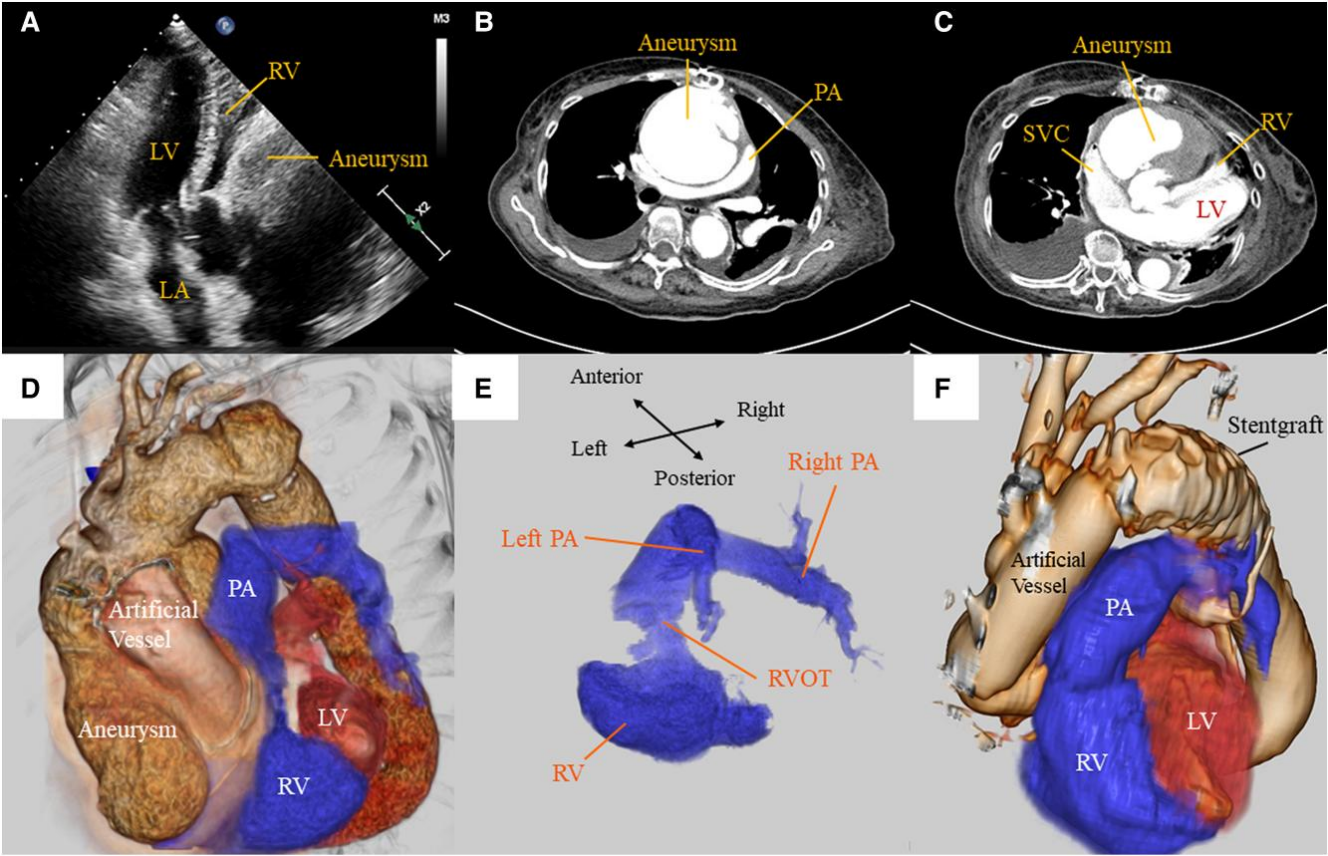


## Supplementary Material

ytae024_Supplementary_Data

## Data Availability

The data underlying this article are available in the article and in its online Supplementary material.

